# The preliminary comparative results between Covid-19 and non-Covid-19 patients in Western China

**DOI:** 10.1186/s12879-020-05680-6

**Published:** 2020-12-09

**Authors:** Yanzi Li, Hongxia Li, Jianfeng Han, Lin Yang

**Affiliations:** 1 Department of Medical Administration, First Affiliated Hospital of Xi’an Jiaotong University, Xi’an, China; 2 Department of Administrative Office, First Affiliated Hospital of Xi’an Jiaotong University, Xi’an, China; 3 Department of Surgery, First Affiliated Hospital of Xi’an Jiaotong University, Xi’an, China

**Keywords:** Coronavirus, Covid-19, Pneumonia, Epidemiology, Comparative results

## Abstract

**Background:**

This study aims to investigate the comparative clinical characteristics of Covid-19 and non-Covid-19 patients.

**Methods:**

Fifteen Covid-19 and 93 non-Covid-19 patients were included in RNA testing. All epidemiological and clinical data were collected and analyzed, and then comparative results were carried out.

**Results:**

Covid-19 patients were older (46.40 ± 18.21 years vs 34.43 ± 18.80 years) and had a higher body weight (70.27 ± 10.67 kg vs 60.54 ± 12.33 kg, *P* < 0.05). The main symptoms that were similar between Covid-19 and non-Covid-19 patients, and Covid-19 patients showed a lower incidence of sputum production (6.67% vs 45.16%, *P* < 0.01) and a lower white-cell count (4.83 × 10^9^/L vs 7.43 × 10^9^/L) and lymphocyte count (0.90 × 10^9^/L vs 1.57 × 10^9^/L, *P* < 0.01). Although there were no differences, C-reactive protein and interleukin-6 were elevated in Covid-19 patients. The sensitivity and negative predictive value of CT images were 0.87 and 0.97, respectively. Covid-19 patients showed a higher contact history of Wuhan residents (80% vs 30.11%) and higher familial clustering (53.33% vs 8.60%, *P* < 0.001). Covid-19 patients showed a higher major adverse events (ARDS, 13.33%; death, 6.67%; *P* < 0.05).

**Conclusion:**

Our results suggested that Covid-19patients had a significant history of exposure and familial clustering and a higher rate of severe status; biochemical indicators showed lymphocyte depletion.

## Background

Since the first 4 cases of coronavirus pneumonia were reported in late December 2019 in Wuhan, China, an outbreak of the 2019 coronavirus disease (Covid-19) has been reported in all areas of China and dozens of other countries [1, 2]; this new virus was identified as the seventh coronavirus using deep gene sequence analysis [3]. By February 27, 2020, 78,630 people were infected with new coronavirus pneumonia, of which 2747 died. Similar to severe acute respiratory syndrome coronavirus (SARS-CoV) and Middle East respiratory syndrome coronavirus (MERS-CoV) with high mortality, Covid-19 is causing serious disease and global health concerns [4–6]. Although several reports have revealed the epidemiological and clinical characteristics of Covid-19 [1, 7], there are currently no reports on the clinical features of Covid-19 in Western China, and no literature on the clinical differences between Covid-19 and non-Covid-19 patients. Thus, analyzing the clinical characteristics of these two groups of patients will play an important role in the treatment of the disease. We carried out a retrospective study to elucidate the comparative results between Covid-19 and non-Covid-19patients.

## Methods

### Patients

There were 108 patients with pneumonia of unknown cause who were admitted to the First Affiliated Hospital of Xi’an Jiaotong University between January20, 2020 and February 26, 2020. According to the guidelines for severe acute respiratory syndrome coronavirus 2 (SARS-CoV-2) infection from the National Health Commission of the People’s Republic of China, throat swab samples of all suspected patients were collected to detect virus titers of Covid-19 via reverse transcription polymerase chain reaction (gold standard); in total,15 patients were confirmed to have Covid-19, and 93 patients were not infected with SARS-CoV-2 (defined as “non-Covid-19 patients”) by Xi’an CDC (Center for Disease Control and Prevention). All patients received computed tomography (CT) scans of the chest and biochemical tests of blood samples. This study was approved by the local ethics committee of the First Affiliated Hospital of Xi’an Jiaotong University, in order to avoid the potential cross-infection, verbal informed consent was obtained from all patients or family members, and the text of the patient’s verbal consent was recorded by the investigator.

### Quantitative reverse transcription polymerase chain reaction

The respiratory tract and blood specimens were collected from the patients at various time points after hospitalization. Viral ribonucleic acid (RNA) were extracted and tested by using the RNA Viral Kit (Daangene, Guangzhou, China) at the Xi’an (Shaanxi, China) CDC, and quantitative reverse transcription polymerase chain reaction was performed using the specific primers and probes of Covid-19, as recommended by the China CDC (http://ivdc.chinacdc.cn/kyjz/202001/t20200121_211337.html?from=timeline&isappinstalled=0). Conditions for the amplifications were 50 °C for 15 min, 95 °C for 15 min, followed by 45 cycles of 94 °C for 15 s and 55 °C for 45 s. A cycle threshold value (Ct value) less than 37 were defined as a positive test (this result is considered as the gold standard), and a Ct value greater than 40 was defined as a negative test. Specimens with a Ct value greater than 37 were repeated and were considered positive if repeated results were the same as the primary result with a Ct value between 37 and 40.

### Clinical procedure

The clinical charts, records, laboratory items, and chest CT scans were collected from all patients with and without a laboratory-confirmed SARS-CoV-2 infection who were reported by the local CDC authority. The CT findings criteria are defined as the interim guidance from the National Health Commission of China and previous literature [8–10]. The epidemiological and symptom data were obtained from the patients or families via direct communication. Clinical, biochemical and radiological characteristic data were obtained from electronic medical records, and these data were collected from the clinical information obtained during the same period of the RNA test.

Antibiotics (orally or intravenously) and lopinavir/ritonavir (250 mg twice daily) were empirically administered. Thymalfasin (1.6 mgdaily) and interferon a-2b (aerosolized inhalation twice daily) were used for therapy. Acute respiratory distress syndrome (ARDS) and shock were defined according to the interim guidance of the WHO for novel coronavirus [11]. Hypoxemia was defined as in previous reports [12, 13]. Oxygen support (nasal cannula and invasive mechanical ventilation) was administered to patients according to the severity of hypoxemia. Repeated tests for Covid-19 were performed in patients with a SARS-CoV-2 infection to show viral clearance before hospital discharge or discontinuation of isolation, the two consecutive RNA tests of sputum and throat swabs were negative, and then the RNA test was still negative at 2 weeks isolation after discharge, which was considered as the complete viral clearance.

### Clinical classification and criteria discharge

The clinical classification of Covid-19 was defined previously by the National Health Commission of China [8] and includes four types: mild, common, severe and critical; mild and common types were defined as non-severe classification; severe and critical types were defined as severe classification. Patients who meet the following four criteria are judged to have recovered and can be released and discharged. Body temperature returned to normal for more than 3 days, respiratory symptoms disappeared and relieved, imaging changes basically disappeared, and RNA test results were negative at two consecutive times (sampling time interval of at least once per day).

### Statistical analysis

All data were analyzed using SPSS v. 11.0 (SPSS, Chicago, IL, USA), and *P* < 0.05 was considered significant. Categorical data were analyzed using the chi-squared test, Fisher’s exact test or analysis of variance, and continuous data were first tested for normality. Normally distributed data are presented as the means (SD), and hypothesis significance testing was performed with paired and unpaired t-tests.

## Results

### The demographic characteristics

Fifteen Covid-19 (13 patients were diagnosed as Covid-19 with the two times positive RNA tests, and 2 patients were diagnosed with Covid-19 after 4 times RNA tests) and 93 non-Covid-19 patients were included in RNA testing in this study (Table 1). Males accounted for 60% of Covid-19 and 65.9% of non-Covid-19 patients; however, the mean age of patients was higher in Covid-19 patients (46.40 ± 18.21 years vs 34.43 ± 18.80 years, *P* = 0.024), and the body weight was higher in Covid-19 patients than in non-Covid-19 patients (70.27 ± 10.67 kg vs 60.54 ± 12.33, *P* = 0.019). In total,15 Covid-19 (100%) and 77 non-Covid-19 (82.8%) patients were adults. Although there was no significant difference in the comorbidities between the two groups, it appears that there was more comorbidity in Covid-19 patients.

**Table 1:** The demographic characteristics between Covia-19 and non-Covid-19

n (%)	Covid-19 (*n* = 15)	Non-Covid-19 (*n* = 93)	*P* value
Gender (M)	9 (60)	61 (65.59)	0.67
Age (years)	46.40 ± 18.21	34.43 ± 18.80	0.024
Weight (Kg)	70.27 ± 10.67	60.54 ± 12.33	0.019
Adults	15 (100)	77 (82.80)	0.18
Comorbidities			0.10
Hypertension	3 (20)	6 (6.45)	
Diabetes	1 (6.67)	4 (4.30)	
Chronic kidney disease	1 (6.67)	1 (1.08)	
Stroke	1 (6.67)	1 (1.08)	
Ventilation	1 (6.67)	0	
Epilepsy	0	2 (2.15)	
Gallstones	0	2 (2.15)	
Coronary atherosclerotic disease	0	2 (2.15)	
Tuberculosis	0	1 (1.08)	
Ovarian cancer	0	1 (1.08)	
Neurological headache	0	1 (1.08)	
Polymyositis	0	1 (1.08)	
Bronchitis	0	2 (2.15)	

*P* value, compared with non-Covid-19

### The clinical characteristics

All clinical data are shown in Table 2. The most common symptoms were fever, which accounted for 93.33% of Covid-19 and 83.87%of non-Covid-19 patients, but there was no significant difference in body temperature between the two groups. Cough was seen in 6 (40%) Covid-19 and 47 (50.54%) non-Covid-19 patients; however, the Covid-19 patients showed a lower incidence of sputum production (6.67% vs 45.16%, *P* = 0.005). There were no differences in the incidence of sore throat (6.67% vs 22.58%), headache (26.67% vs 11.83%), myalgia or fatigue (33.33% vs 25.81%), dyspnea (6.67% vs 9.68%) or intestinal symptoms (6.67% vs 2.15%) between Covid-19 and non-Covid-19 patients, and catarrhal symptoms were only confirmed in patients with non-Covid-19 (10.75%). There wasa higher severe classification in Covid-19 patients than in non-Covid-19 (26.67% vs 4.30%, *P* = 0.01).

**Table 2:** The clinical characteristics between Covia-19 and non-Covid-19

n (%)	Covid-19 (*n* = 15)	Non-Covid-19 (*n* = 93)	*P* value
Fever (Yes)	14 (93.33)	78 (83.87)	0.57
Body temperature (°C)	38.18 ± 0.64	38.27 ± 0.78	0.68
Cough	6 (40)	47 (50.54)	0.45
Sputum production	1 (6.67)	42 (45.16)	0.005
Sore throat	1 (6.67)	21 (22.58)	0.28
Headache	4 (26.67)	11 (11.83)	0.25
Catarrhal symptoms	0	10 (10.75)	0.39
Myalgia or fatigue	5 (33.33)	24 (25.81)	0.77
Dyspnea	1 (6.67)	9 (9.68)	0.39
Intestinal symptoms	1 (6.67)	2 (2.15)	0.89
Clinical classification			0.01
Non-severe	11 (73.33)	89 (95.70)	
Severe	4 (26.67)	4 (4.30)	

*P* value, compared with non-Covid-19

### The clinical laboratory results

A blood sample test was conducted for all patients, and the results are listed in Table 3. Compared with non-Covid-19 patients, patients with Covid-19 showed a lower white-cell count (4.83 × 10^9^/L vs 7.43 × 10^9^/L, *P* = 0.002), lymphocyte count (0.90 × 10^9^/L vs 1.57 × 10^9^/L, *P* = 0.002) and platelet count (175.93 × 10^9^/L vs 244.03 × 10^9^/L, *P* = 0.01). The other items in routine blood tests were similar in both groups. The serum chloride level was lower in Covid-19 patients than that in non-Covid-19 patients, while the carbon dioxide binding rate and blood glucose level were higher in Covid-19 patients. The level of alkaline phosphatase was higher in Covid-19 patients than that in non-Covid-19 patients, but at a statistically critical value (*P* = 0.05). There were no significant differences in other indicators of liver function, kidney function, electrolytes, coagulation function, and myocardial enzymes between the two groups. No differences were confirmed in the inflammatory factors, C-reactive protein and interleukin-6, between the two groups; however, the value of both factors was higher in patients with Covid-19.

**Table 3:** Clinical laboratory results between Covid-19 and non-Covid-19

Items	Reference range	Covid-19 (*n* = 15)	Non-Covid-19 (*n* = 93)	*P* value
White-cell count (10^9^/L)	3.5–9.5	4.83 ± 2.13	7.43 ± 3.07	0.002
Red-cell count (10^12^/L)	4.3–5.8	4.81 ± 0.62	4.66 ± 0.60	0.37
Neutrophil count (10^9^/L)	1.8–6.30	3.55 ± 2.40	5.11 ± 2.96	0.06
Lymphocyte count (10^9^/L)	1.1–3.2	0.90 ± 0.40	1.57 ± 0.82	0.002
platelet count (10^9^/L)	125–350	175.93 ± 73.43	244.03 ± 98.20	0.01
Hemoglobin (g/L)	130–175	140.40 ± 20.80	137.95 ± 18.10	0.63
Sodium (mmol/L)	137–147	137.33 ± 2.79	139.01 ± 4.54	0.19
Potassium (mmol/L)	3.5–5.3	3.92 ± 0.58	4.18 ± 0.65	0.18
Chloride (mmol/L)	96–108	101.67 ± 8.87	106.52 ± 6.14	0.027
Calcium (mmol/L)	2.11–2.52	2.12 ± 0.31	2.01 ± 0.19	0.14
Carbon dioxide (mmol/L)	22–29	25.23 ± 2.65	21.46 ± 3.18	< 0.001
Anion gap (mmol/L)	–	17.45 ± 6.70	18.44 ± 5.07	0.56
Glucose (mmol/L)	3.9–6.1	7.08 ± 2.38	5.50 ± 1.29	< 0.001
Blood urea nitrogen (mmol/L)	3.6–9.5	4.58 ± 1.70	5.75 ± 8.80	0.61
Creatinine (umol/L)	57–111	55.27 ± 17.45	82.44 ± 140.55	0.46
Total protein (g/L)	65–85	69.32 ± 6.74	69.82 ± 7.57	0.81
Albumin (g/L)	40–55	40.42 ± 5.10	41.45 ± 6.78	0.58
Total bilirubin (umol/L)	3.4–17.1	13.30 ± 5.78	10.43 ± 6.79	0.13
Alanine aminotransferase (U/L)	9–50	32.33 ± 20.17	26.96 ± 22.84	0.39
Aspartate aminotransferase (U/L)	15–40	30.60 ± 15.54	28.15 ± 14.45	0.55
Alkaline phosphatase (U/L)	45–125	60.53 ± 16.36	89.06 ± 55.01	0.05
Creatine kinase (U/L)	50–310	218.93 ± 542.03	136.96 ± 420.01	0.50
Lactate dehydrogenase (U/L)	120–250	257.0 ± 168.48	253.92 ± 271.77	0.96
Prothrombin time (s)	11–14	13.25 ± 0.44	13.96 ± 1.35	0.06
Fibrinogen (g/L)	2–4	4.05 ± 0.98	4.20 ± 1.92	0.78
Activated partial thromboplastin time(s)	28–43.5	38.79 ± 4.89	39.02 ± 6.95	0.91
D-dimer (mg/L)	0–1	1.77 ± 3.16	1.78 ± 2.96	0.95
C-reactive protein (mg/L)	0–10	28.71 ± 54.49	19.89 ± 33.40	0.39
Procalcitonin (ng/mL)	< 5	0.13 ± 0.29	0.12 ± 0.20	0.87
Interleukin-6 (pg/mL)	0–7	13.47 ± 18.13	7.58. ± 13.25	0.51

*P* value, compared with non-Covid-19

**Table 4:** The epidemiological and clinical outcome between Covia-19 and non-Covid-19

n (%)	Covid-19 (*n* = 15)	Non-Covid-19 (*n* = 93)	*P* value
Exposure in Wuhan	12 (80)	28 (30.11)	< 0.001
Family cluster	8 (53.33)	8 (8.60)	< 0.001
Computed tomography of chest			< 0.001
Inflammatory lesions	2 (13.33)	52 (55.91)	
Viral lesions	13 (86.67)	29 (31.18)	
None	0	12 (12.91)	
Recover after therapy	11 (73.33)	88 (94.62)	0.024
Viral clearance	11 (78.57)	Null	Null
Positive again	3 (20)	Null	Null
Death	1 (6.67)	0	0.29
ARDS	2 (13.33)	0	0.011
Hospital stay (days)	18.60 ± 6.05	5.73 ± 3.72	< 0.001

*P* value, compared with non-Covid-19

### The epidemiological characteristics

In this study, 12 Covid-19 (80%) and 28 non-Covid-19 (30.11%) patients had contact with Wuhan residents (Table 4, *P* < 0.001), and the odds ratio (OR) was 9.71(95% confidence interval (CI), 2.93–32.26). Eight patients in both groups had a familial cluster (53.33% vs 8.60%, *P* < 0.001), and the OR was 12.14 (95% CI, 3.72–39.25).

### Outcome

**Fig. 1:**
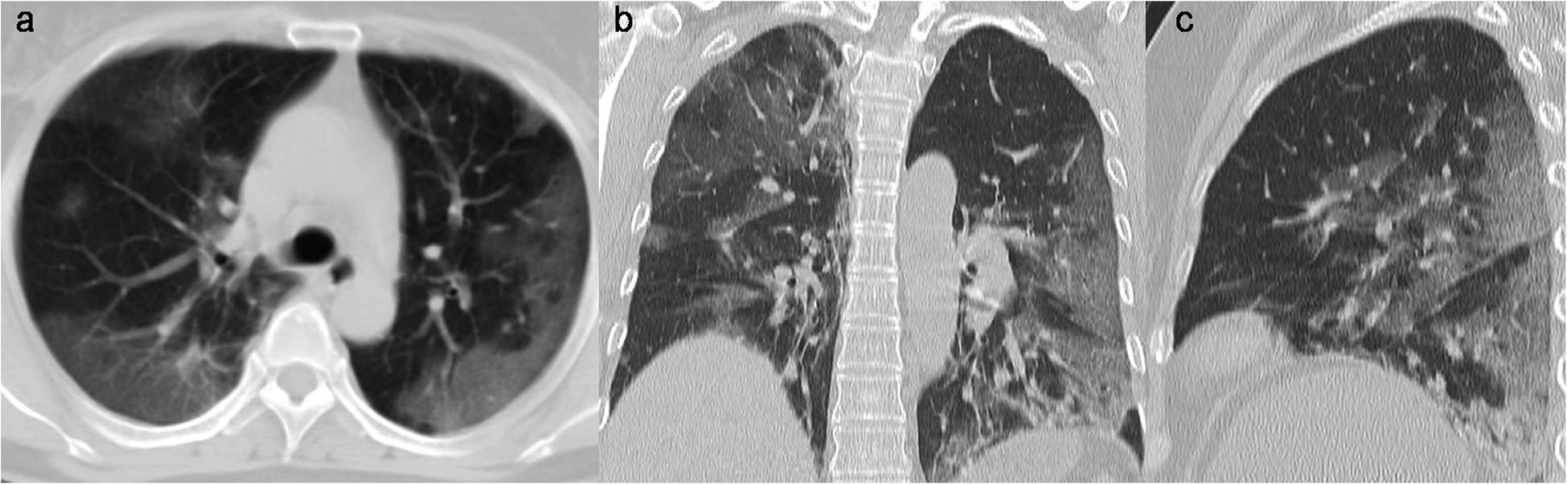
The chest CT images of the Covid-19 patient. CT images showed scattered exudative lesions in lungs, large ground-glass lesions, and partial consolidation of the lesions in the lower lobe of the left lung; **a**) transverse section; **b**) coronal section; **c**) median sagittal section

The chest CT of Covid-19 patients mainly showed multiple small patchy shadows and interstitial changes in both lungs in the early stage, and then developed multiple flaky ground glass shadows in both lungs, accompanied by partial consolidation of lung lobes (Fig. 1). The CT images showed viral lesions in 13 Covid-19 patients and 29 non-Covid-19 patients (86.67% vs 31.18%, *P* < 0.001, Table 4). When compared with the RNA test, the sensitivity and negative predictive value of the Ct scan were 0.87 and 0.97, respectively. During the process, 11 Covid-19 patients (73.33%) and 88 non-Covid-19 patients (94.62%) recovered and were discharged (*P* = 0.024), and the viral clearance of the Covid-19 were 78.57%. Four patients with severe clinical classification were admitted in intensive care unit, and 2 Covid-19 patients (13.33%) with severe classification developed ARDS and were treated with mechanical ventilation, and one of these patients died (6.67%). The hospital stay of patients with Covid-19 was longer than that of non-Covid-19 patients (18.60 ± 6.05 days vs 5.73 ± 3.72 days, *P* < 0.001). Three patients had a positive RNA test after two consecutive negative RNA tests and then continued to receive treatment, and then.

## Discussion

Currently, Covid-19 is the fourth large-scale outbreak of coronavirus after SARS and MERS, affecting China and dozens of other countries and seriously affecting global health security [1, 2, 14, 15]. According to statistics, China may lose $62 billion in the first quarter of 2020, while world losses may be over $280 billion [16, 17]. Thus, comparing the clinical differences between Covid-19 and non-Covid-19 patients is very important for disease prevention and control. In this study, the patients with Covid-19 showed advanced age and more comorbidity. This finding indicates that Covid-19 may be more susceptible to older people with more comorbidity, and the clinical manifestations may be more serious and lead to adverse outcomes. Four patients developed a severe classification (26.67%), ARDS occurred in two elderly patients (13.33%) with Covid-19, and one of these two patients died in this study. These findings were consistent with the earlier epidemiological conclusions about Covid-19 [7, 18].

Fever and dry cough were the most common symptoms for the patients with and without a SARS-CoV-2 infection; however, sputum production was confirmed more in non-COVID-19 patients (45.16% vs 6.67%). In addition, few patients showed upper respiratory tract signs and symptoms, including sore throat (6.67%) and catarrhal symptoms (0%). These findings suggested that Covid-19 exhibits similar clinical characteristics to SARS and MERS [15, 19]. The catarrhal symptoms were only present in non-Covid-19 patients (10.75%); in addition, intestinal symptoms (6.67% vs 2.15%) were very low in both Covid-19 and non-Covid-19 patients, whereas the intestinal symptoms (20–25%) were more confirmed in patients with MERS or SARS [20].

People with a history of exposure to Wuhan residents had a higher incidence of Covid-19 (OR, 9.71; 95% CI, 2.93–32.26) and familial clustering (OR, 12.14;95% CI, 3.72–39.25), which means that local cases were still dominated by Wuhan imports, and the new coronavirus has a dramatic human-to-human transmission. However, 20% of patients with Covid-19 did not have any contact history, which meant that Covid-19 has other pathogenesis and transmission routes. In addition, patients without obvious contact history and disease transmission may be the focus of prevention and control in the future.

Our study revealed that the white blood cell and lymphocyte countswere significantly lower in patients with Covid-19. These results suggested that Covid-19 might mainly act on lymphocytes and generate a series of pro-inflammatory cytokines and that a higher level of cytokine storms was associated with the severity of disease and a poor prognosis [1, 21–23]. However, SARS-CoV-2 infection also had higher levels of anti-inflammatory cytokines, including T helper 2 and interleukin-10, making it different from SARS [23]. A previous report suggested that the damage and consumption of T lymphocytes may be an important factor leading to the rapid deterioration of SARS and MERS; perhaps Covid-19 has the same mechanism [24]. Although the difference has not been confirmed, patients with Covid-19 showed higher C-reactive protein and interleukin-6, which might be correlated with the limitation of, sample size; however, the higher inflammatory reaction was confirmed by a previous report [1, 22, 25].

Because no effective antiviral drug has been identified, the treatment of Covid-19 patients is based on the experience of treating SARS and MERS [19, 20, 26]. Interferon a-2b, thymalfasin and antiviral drug lopinavir/ritonavir were used for the treatment of patients in this study. Our results showed that the therapies for non-severe patients were effective, the viral clearance rate was 78.57% when the patients released form 2 weeks isolation, but it is still not ideal for severe patients; life support therapy and mechanical ventilation may be effective in severe patients, but the mortality rate in severe cases remains high [1, 22]. Further research is needed on related drugs and vaccines for the purpose of controlling and treating Covid-19.

CT imaging is very important for the diagnosis and treatment of patients with Covid-19, especially where RNA testing is inconvenient. In this study, the sensitivity and negative predictive value of CT images were 0.87 and 0.97, respectively. The CT images indicated that multifocal peripheral ground-glass or mixed opacity with predominance in the lower lung is highly suspicious of Covid-19 [27]. Although the RNA test was negative, the obvious CT findings may indicate the presence of a SARS-CoV-2 infection; thus, repeated RNA tests are needed to be performed in patients with typical symptoms and exposure; these data are consistent with previous studies [9, 10]. In our study, 3 patients had a positive RNA test result after therapy after two consecutive negative results; therefore, RNA tests and CT scans during follow-up may play an important role in the prognosis examination of patients with Covid-19.

The limitations of this study are as follows. First, this study has a small sample size, as only 15 Covid-19 patients and 95 non-Covid-19 patients were included; it is better to have more patients from Western China included in this study to more clearly understand the differences between Covid-19 and non-Covid-19 patients. Second, due to time constraints, the information of the whole clinical process is not included, which might cause bias of selection. Third, this study is not a randomized clinical trial, and the conclusions need to be confirmed in the future.

## Conclusions

When compared with non-Covid-19 patients, patients with Covid-19 had a significant history of exposure and familial clustering onset and a higher rate of severe status; the clinical biochemical indicators showed significant lymphocyte depletion and elevated inflammatory factors.

## Data Availability

The datasets used and analyzed during this study available from the corresponding author on reasonable request. We confirmed that these patients have not been reported in any other submission by all authors or anyone else.

## Abbreviations

Covid-19: Coronavirus disease-2019
SARS-CoV: Severe acute respiratory syndrome coronavirus
SARS-CoV-2: Severe acute respiratory syndrome coronavirus 2
MERS-CoV: Middle East respiratory syndrome coronavirus
CDC: Center for disease control and prevention
CT: Computed tomography
RNA: Ribonucleic acid
ARDS: Acute respiratory distress syndrome
OR: Odds ratio
CI: Confidence interval
